# MRI for differentiating ovarian endometrioid adenocarcinoma from high-grade serous adenocarcinoma

**DOI:** 10.1186/s13048-015-0154-2

**Published:** 2015-04-30

**Authors:** Hai Ming Li, Jin Wei Qiang, Gan Lin Xia, Shu Hui Zhao, Feng Hua Ma, Song Qi Cai, Feng Feng, Ai Yan Fu

**Affiliations:** Department of Radiology, Jinshan Hospital, Shanghai Medical College, Fudan University, 1508 Longhang Road, Shanghai, 201508 China; Department of Radiology, Nantong Cancer Hospital, Nantong University, 30 North Tongyang Road, Tongzhou District, Nantong, Jiangsu 226361 China; Department of Radiology, Xinhua Hospital, Shanghai Medical College, Jiaotong University, 1665 Kongjiang Road, Shanghai, 200092 China

**Keywords:** Ovary, Endometroid carcinoma, High-grade serous carcinoma, Magnetic resonance imaging, Diffusion-weighted imaging

## Abstract

**Purpose:**

To investigate magnetic resonance imaging (MRI) features for differentiating ovarian endometrioid adenocarcinoma (OEC) from high-grade serous adenocarcinoma (HGSC).

**Materials and methods:**

Twenty-three patients with 25 OECs and 93 patients with 139 HGSCs confirmed by surgery and pathology underwent conventional MRI and diffusion-weighted imaging (DWI). The MRI features of the tumors, including laterality, size, shape, configuration, signal intensity, ADC value of solid component, enhancement, ascites, synchronous primary cancer (SPC) of the ovary and endometrium, and clinical stage, were evaluated and compared between two groups.

**Results:**

The following characteristics were significantly more common for OECs than HGSCs: unilateral (91.3% vs 50.5%, P < 0.001), larger mass (80.0% vs 48.2%, P = 0.005), round or oval shape (64.0% vs 17.3%, P < 0.001), mainly cystic with mural nodules or papillary projections (72.0% vs 18.7%, P < 0.001), cystic component with homogeneous iso- or hyperintensity on T1WI (82.6% vs 4.3%, P < 0.001), moderate enhancement (52.0% vs 26.6%, P = 0.011), no or mild ascites (91.3% vs 57.0%, P = 0.002), and SPC (43.5% vs 4.3%, P < 0.001). The ADC value of the solid component was higher in OECs (0.979 ± 0.197 × 10^−3^ mm^2^/s) than in HGSCs (0.820 ± 0.112 × 10^−3^ mm^2^/s) (P = 0.002). When a mainly cystic mass with mural nodules or papillary projections was associated with any one of homogeneously iso- or hyperintense cystic component on TIWI, a relatively higher ADC value and SPC, the sensitivity, specificity, accuracy, and positive and negative predictive values for characterizing OEC were 87.0%, 93.5%, 92.2%, 76.9%, and 96.7%, respectively.

**Conclusions:**

Conventional MRI combining DWI is helpful for differentiating OECs from HGSCs.

## Background

When an adnexal mass is suspected to be an epithelial ovarian carcinoma by magnetic resonance imaging (MRI), the primary concern of clinicians is that the patient has a high-grade serous adenocarcinoma (HGSC), which is the most frequent epithelial carcinoma and is typically aggressive clinically [1]. However, ovarian endometrioid carcinoma (OEC), the second most common type of epithelial carcinoma, accounts for 10% of all epithelial ovarian malignancies, shares similar morphologic features to HGSC [2]. Previous studies have indicated that there are different risk factors, origins, genetic alterations, biological behaviors, clinicopathological characteristics and chemotherapy sensitivities between OEC and HGSC. Clincally, patients with OECs are more likely to have early stage disease and generally have a favorable prognosis [3-6]. However, few studies have focused on the imaging of OEC [7]. As a result, the imaging features of OEC have not been completely identified. Furthermore, conventional MRI and diffusion weighted imaging (DWI) have not been investigated for their ability to differentiate between OEC and HGSC. Preoperative imaging differentiation of OEC from HGSC will be helpful for the management of patient with OEC, because a conservative fertility-sparing surgery can be considered for patients with early-stage OEC who wish to preserve fertility [8]. Therefore, this retrospective study evaluated conventional MRI and DWI for distinguishing OEC from HGSC to improve the preoperative characterization and surgical planning of these two distinctive types of ovarian cancers.

## Methods

### Clinical data

The institutional ethical board (Jinshan Hospital, Shanghai Medical College, Fudan University) approved this retrospective study, and informed consent was waived. Between September, 2010 and October, 2014, we searched for the data of patients with OEC at our hospitals information system and picture achiving and communication system. A total of 25 patients with OEC confirmed by surgery and pathology were found. We excluded 2 patients with metastatic OEC. The remaining 23 patients with 25 OECs were reviewed in this study. The mean age of the patients was 54 ± 9 years (range, 32–81 years). As a comparison, 93 patients with 139 HGSCs were served as a control group from 126 surgically and pathologically confirmed cases of HGSC at the same period and database. We excluded 15 patients who received chemotherapy before MR scanning, 8 patients who were performed on a 3.0 T MR scanner, 6 patients with poor image quality and 4 patients without the administration of intravenous contrast. Their mean age was 55 ± 9 years (range, 35–78 years), which was not significantly different from the mean age of patients with OEC (P = 0.508). The diagnosis of SPC was established according to the pathological criteria proposed by Singh [9]. Twelve patients presented with abdominal pain and swelling; eight patients presented with vaginal bleeding; two patients were asymptomatic and were diagnosed during a routine physical examination; and the last patient presented with an abdominal mass. All patients underwent surgery within 2 weeks after completing the MRI scan.

### MRI scanning

MR imaging was performed using a 1.5-T scanner (Avanto or Espree, Siemens, Erlangen, Germany) with a phased-array abdominal coil. The patients laid in a supine positon and breathed freely during acquisition. The sequences were obtained as follows: axial spin echo (SE) T1-weighted imaging (T1WI) [time of repetition (TR)/time of echo (TE), 340 ms/10 ms]; axial turbo SE T2-weighted imaging (T2WI) with and without fat saturation (TR/TE, 8000 ms/83 ms and 4000 ms/98 ms, respectively); and sagittal and coronal turbo SE T2WI (TR/TE, 8000 ms/98 ms). Axial DWI (19 patients with 20 OECs and 83 patients with 124 HGSCs) was obtained with echo planar imaging (TR/TE, 3200 ms/83 ms) and b factors of 0 and 1000 s/mm^2^. Contrast-enhanced flash 2D T1WI with fat saturation (TR/TE, 196 ms/2.9 ms) was performed in the axial, sagittal and coronal planes immediately after the intravenous administration of Gadopentetate dimeglumine (Magnevist; Bayer Schering, Guangzhou, China) at a dose of 0.2 mmol/kg of body weight and a rate of 2–3 ml/s. The scanning parameters were as follows: 5-mm slice thickness, 1.2-mm gap, 256–320 × 256–320 matrix, 250–296 mm × 250–340 mm field of view and four excitations. The scanning range was from the inferior pubic symphysis to the renal hilum and extended beyond the dome of the tumor in cases with huge masses.

### Image analysis

The MR images were reviewed independently by two radiologists (H.M.L and J.W.Q) with 7 years and 30 years of experience in abdominal imaging, respectively, and were blinded to the original reports (radiology, surgery and pathology). Any discrepancies were resolved by consensus. The following features of the tumors were evaluated: (a) uni- or bilaterality (both ovaries having similarly sized tumors, which indicates the simultaneous development of primary malignancies), size and shape; (b) mass configuration (mainly cystic, less than one-third solid component; mixed cystic-solid, one- to two-thirds solid component; and solid, more than two-thirds solid component); (c) signal intensity (hypo-, iso-, and hyperintensity, referring to the signal of the outer myometrium in solid components; to the signals of muscle and iliac marrow in cystic components on T1WI and T2WI; and to the signals of small intestine and iliac vessel on DWI and ADC maps, respectively); (d) enhancement (mild, moderate or marked by referencing those of the junctional zone and outer myometrium); (e) amount of ascites (none, mild, moderate, and severe) (f) associated findings (uterine endometrial carcinoma); and (g) apparent diffusion coefficient (ADC) value as measured on ADC maps, a circular region of interest (ROI) of at least 1 cm^2^ was placed at targeted areas with the possibly lowest ADC values in the solid components of the tumor, by referring to conventional MR imagings and avoiding areas such as haemorrhage, necrosis and major vascular structures. At least three measurements were obtained and averaged.

### Statistical analysis

SPSS 16.0 for Windows (SPSS Inc., Chicago, IL, USA) was used for the statistical analysis. The differences between OECs and HGSCs in laterality, shape, mass configuration, signal intensity, enhancement, ascites, SPC, and clinical stage were compared using a Pearson chi-square test. The differences in age, mass size, and ADC values between the two groups were compared using the two independent-sample Student *t*-test. A P-value less than 0.05 was considered statistically significant. Receiver operating characteristic (ROC) curve analysis was used to determine a cut-off value for differentiating OECs and HGSCs.

## Results and discussion

The maximum diameters of the OECs ranged from 3.7 to 22.5 cm (mean 11.1 ± 4.9 cm), and those of the HGSCs ranged from 2.5 to 16.8 cm (8.1 ± 3.4 cm) (P = 0.005). The median size of the tumors was 9.9 cm in OECs versus 7.6 cm in HGSCs. A bilateral mass was found in 2 OEC patients and 46 HGSC patients (P < 0.001). According to the International Federation of Gynecology and Obstetrics (FIGO) staging system, 19 patients with OECs were at stage I (82.6%), 3 were at stage II (13.0%), and 1 was at stage III (4.4%). Pathologically, 14 OECs were grade 1, 8 were grade 2, and 1 was grade 3. For the HGSC group, 6 patients were at stage I (6.4%), 9 were at stage II (9.7%), 73 were at stage III (78.5%), and 5 was at stage IV (5.4%). There was a statistically significant difference in the number of patients at different stages of disease between the two groups (P < 0.000).

**Figure 1:**
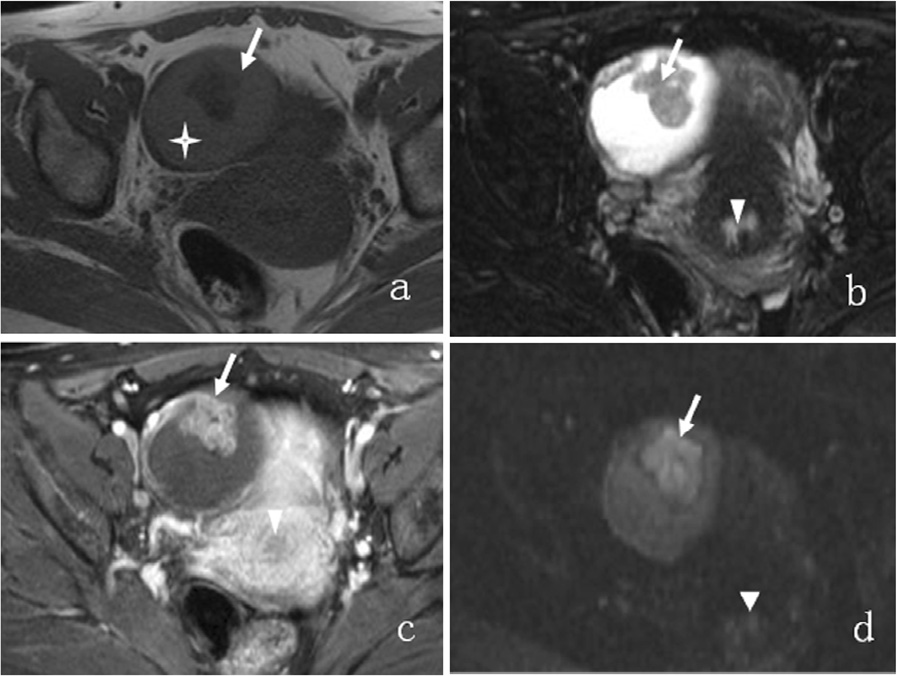
A 50-year-old woman with right-sided OEC. The tumor appears as a mainly cystic mass with a large mural nodule. The cystic component (crossstar) shows slight hyperintensity on axial T1WI **(a)**, homogeneous hyperintensity on axial T2WI with fat suppression **(b)**, no enhancement on contrast-enhanced T1WI with fat suppression **(c)**, isointensity on DWI **(d)**. The mural nodule (arrow) demonstrates isointensity **(a)**, heterogenous hyperintensity **(b)**, marked enhancement **(c)** and hyperintensity **(d)** with an ADC value of 1.030 × 10^−3^ mm^2^/s, respectively. Synchronous uterine endometrial carcinoma (arrowhead) shows mild enhancement on contrast-enhanced T1WI and hyperintensity on DWI.

**Figure 2:**
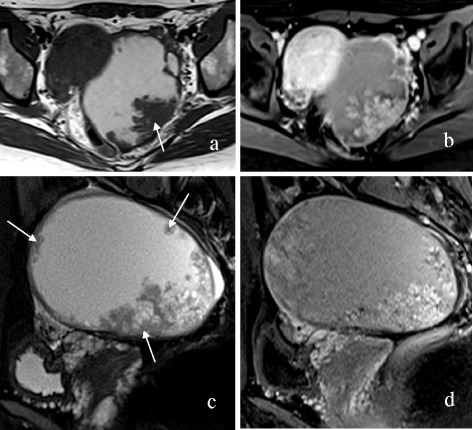
A 32-year-old woman with left-sided OEC. Axial and sagittal T2WI **(a, c)** demonstrate a mainly cystic mass with multiple mural nodules (arrows). Axial and sagittal contrast-enhanced T1WI with fat suppression **(b, d)** show that the nodules are moderately enhanced.

**Figure 3:**
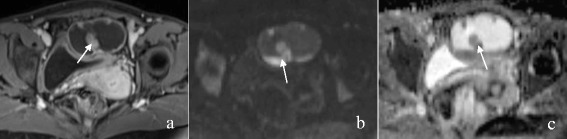
A 53-year-old woman with left-sided OEC. Contrast-enhanced T1WI with fat suppression **(a)** demonstrates a mainly cystic mass with mural nodules (arrow), which show a marked enhancement **(a)** and a hyperintensity on DWI **(b)** with an ADC value of 1.174 × 10^−3^ mm^2^/s**(c)**.

**Figure 4:**
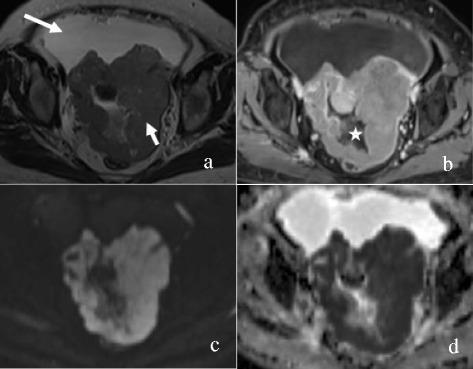
A 67-year-old woman with left-sided HGSC. Axial T2WI **(a)** demonstrates a solid mass (short arrow) with an irregular shape and a large volume of ascites (long arrow). The mass is markedly enhanced with irregular areas of necrosis (asterisk) on contrast-enhanced T1WI with fat suppression **(b)**, hyperintensity on DWI **(c)** and hypointensity on ADC map with an ADC value of 0.682 × 10^−3^ mm^2^/s **(d)**.

**Figure 5:**
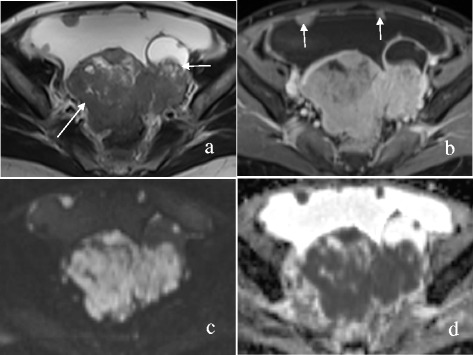
A 57-year-old woman with bilateral HGSC. Axial T2WI **(a)** demonstrates a solid mass (long arrow) in the right-sided ovary and a mixed cystic-solid mass (short arrow) in the left-sided ovary, which have an irregular shape. A large volume of ascites is observed. The solid components of the masses and peritoneal nodules (arrows) are enhanced markedly on contrast-enhanced T1WI with fat suppression **(b)**, hyperintensity on DWI **(c)** and hypointensity on ADC map **(d)** with an ADC value of 0.759 × 10^−3^ mm^2^/s (left) and 0.782 × 10^−3^ mm^2^/s (right).

The MRI features of OEC and HGSC are summarized in Table 1. The mass was round or oval in 16 of 25 (64.0%) OECs versus 24 of 139 (17.3%) HGSCs (P < 0.001). The mass configuration was mainly cystic with mural nodules or papillary projections in 18 of 25 (72.0%) OECs versus 26 of 139 (18.7%) HGSCs (P < 0.001). The signal of the cystic component was homogeneously iso- or hyperintense on TIWI in 19 of 23 (82.6%) OECs versus 3 of 69 (4.3%) HGSCs (P < 0.001) (Figures 1, 2 and 3). The solid component showed a moderate enhancement in 13 of 25 (52.0%) OECs versus 37 of 139 (26.6%) HGSCs (P = 0.011). No or mild ascites were observed in 21 of 23 (91.3%) OECs versus 53 of 93 (57.0%) HGSCs (P = 0.002) (Figures 4 and 5). SPC was observed in 10 of 23 (43.5%) OECs versus 4 of 93 (4.3%) HGSCs (P < 0.001) (Figure 1). Other features, such as the signal intensity of the solid component on T1WI, T2WI, and the signal intensity of the cystic component on T2WI were not significantly different between the two groups (P > 0.05).

**Table 1:** **MRI morphological features of OEC versus HGSC**

**Morphological features**	**OEC (n = 25)**	**HGSC (n = 139)**	**P value**
Laterality^			0.000
Bilateral	2 (8.7%)	46 (49.5%)	
Unilateral	21 (91.3%)	47 (50.5%)	
Shape			0.000
Round/oval	16 (64.0%)	24 (17.3%)	
Lobulated	6 (24.0%)	26 (18.7%)	
Irregular	3 (12.0%)	89 (64.0%)	
Configuration			0.000
Cystic with mural nodules or papilla	18 (72.0%)	26 (18.7%)	
Mixed cystic-solid	5 (20.0%)	43 (31.0%)	
Solid	2 (8.0%)	70 (50.3%)	
SI of solid component on T1WI			0.343
Iso-/hypointense	24 (96.0%)	121 (87.1%)	
Mainly iso- with hyperintense	1 (4.0%)	18 (12.9%)	
SI of solid component on T2WI			0.576
Isointense	3 (12.0%)	9 (6.5%)	
Heterogeneous hyperintense	22 (88.0%)	130 (93.5%)	
SI of cystic component on T1WI			0.000
Homogeneous hypointense	4 (17.4%)	46 (66.7%)	
Homogeneous iso-/hyperintense	19 (82.6%)	3 (4.3%)	
Mainly hypo- with iso-/hyperintense	0	20 (29.0%)	
SI of cystic component on T2WI			0.751
Homogeneous hyperintense	18 (78.3%)	58 (84.1%)	
Mixed signal*	5 (21.7%)	11 (15.9%)	
Enhancement			0.011
Moderate	13 (52.0%)	37 (26.6%)	
Marked	12 (48.0%)	102 (73.4%)	
Ascites^			0.002
None or mild	21 (91.3%)	53 (57.0%)	
Moderate or severe	2 (8.7%)	40 (43.0%)	
SPC^	10 (43.5%)	4 (4.3%)	0.000

SI: signal intensity; *Mixed signal means that two or more signals within the tumors. ^ numbers for 23 patients with OEC and 93 patients with HGSC.

On DWI, the solid component showed hyperintensity in 17 of 20 (85.0%) OECs and isointensity in the remaining 3 (15.0%), while for HGSCs, the solid component showed hyperintensity in 111 of 116 (95.7%) and isointensity in 5 (4.3%) (P = 0.173). The mean ADC value of the solid component was (0.979 ± 0.197) × 10^−3^ mm^2^/s in 20 of 25 OECs versus (0.820 ± 0.112) × 10^−3^ mm^2^/s in 116 of 139 HGSCs (P = 0.002) (Figures 1, 3, 4 and 5). ROC curve analysis yielded an optimal ADC value threshold of 0.971 × 10^−3^ mm^2^/s for differentiating OECs from HGSCs, with a sensitivity of 55.0%, a specificity of 94.0% and an accuracy of 88.2%.

The diagnostic performance of the different MRI features for differentiating OEC from HGSC are listed in Table 2. When a mainly cystic mass with mural nodules or papillary projections was associated with any one of homogeneously iso- or hyperintense cystic component on TIWI, a higher ADC value, and SPC, the sensitivity, specificity, accuracy, positive and negative predictive values for characterizing OEC were 87.0%, 93.5%, 92.2%, 76.9%, and 96.7%, respectively.

**Table 2:** **Diagnostic performance of MRI for differentiating OEC from HGSC**

**MRI features**	**Sensitivity**	**Specificity**	**Accuracy**	**PPV(%)**	**NPV(%)**
Mass size (≥7.8 cm)	80.0(20/25)	51.8(72/139)	56.1(92/164)	23.0(20/87)	93.5(72/77)
Unilateral	91.3(21/23)	49.5(46/93)	57.8(67/116)	30.9(21/68)	95.8(46/48)
Round/Oval shape	64.0(16/25)	82.7(115/139)	79.9(131/164)	40.0(16/40)	92.7(115/124)
Mainly cystic mass	72.0(18/25)	81.3(113/139)	79.9(131/164)	40.9(18/44)	94.2(113/120)
Homogeneous iso-/hyperintensity*	82.6(19/23)	95.7(66/69)	92.4(85/92)	86.4(19/22)	94.3(66/70)
Moderate enhancement	52.0(13/25)	73.4(102/139)	70.1(115/164)	26.0(13/50)	89.5(102/114)
Ascites(none or mild)	91.3(21/23)	43.0(40/93)	52.6(61/116)	28.4(21/74)	95.2(40/42)
SPC	43.5(10/23)	95.7(89/93)	85.3(99/116)	71.4(10/14)	87.3(89/102)
ADC value^	55.0(11/20)	94.0(109/116)	88.2(120/136)	61.1(11/18)	92.4(109/118)

PPV: positive predictive value; NPV: negative predictive value; *the signal intensity of cystic component on T1WI; ^ ADC value of solid component (≥0.971 × 10^−3^ mm^2^/s), ADC values of solid components are measured and averaged in 20 OECs and 116 HGSCs.

During the past decade, there has been strong interest in type-specific treatment of epithelial ovarian carcinoma, and considerable advances have been achieved in the understanding and differentiation of the five types of ovarian cancers (high-grade serous adenocarcinoma, endometrioid carcinoma, clear cell carcinoma, mucinous carcinoma, low-grade serous adenocarcinoma) [10,11]. Ovarian HGSCs and OECs are two of the most frequent types of epithelial ovarian carcinomas. Studies have indicated that most OECs are diagnosed at an early stage, have a low histologic grade, respond well to chemotherapy, and consequently, have a lower recurrence rate and a better survival compared with HGSCs [5]. Furthermore, studies have suggested that the fertility-preserving surgery has a low currence rate and is safe for the patient with stage I epithelial ovarian carcinoma. There is no difference in the overall survival compared with the radical surgery [12-15]. In contrast, patients with HGSC need radical surgical staging and cytoreduction [8]. Therefore, discriminating OEC from HGSC is essential for preoperative surgical planning, especially in stage I patients who wish to preserve fertility and/or female endocrine functions.

The present MRI study showed that OECs were significantly different from HGSCs in laterality, size, shape, configuration, signal intensity, enhancement, ADC value, SPC, clinical stage and ascites. OEC commonly appeared as a large, unilateral, round or oval cystic mass with mural nodules or papillary projections, homogeneous iso- or hyperintensity on T1WI in the cystic component, and moderate enhancement and relatively higher ADC values in the solid component. OEC was commonly associated with SPC and was diagnosed at an early stage. In contrast, HGSC was typically a moderately sized, irregular solid or mixed cystic-solid mass with marked enhancement and a lower ADC value, more common bilaterality and moderate to severe ascites. Although some features had low diagnostic specificity, four features, a cystic mass with mural nodules or papillary projections, homogeneously iso- or hyperintense cystic component on T1WI, higher ADC value and SPC, have a high specificity for OEC. Those four features yielded a sensitivity, specificity, accuracy, positive and negative predictive values for characterizing OEC of 87.0%, 93.5%, 92.2%, 76.9%, and 96.7%, respectively.

Histopathologically, it has been well-established that atypical endometriosis is a precursor lesion for OEC. OEC associated endometriosis is found in 20%-40% of cases [16]. In contrast, most ovarian HGSCs are confirmed to be derived from the tubal intraepithelial lining [17], and only 7% of cases have a history of ovarian endometriosis [18]. In our study, only 13.0% (3/23) of OECs were shown histopathologically to have arisen from an endometriotic cyst, an incidence lower than that of a previous study (33.3%) [7]. A possible explanation for this result is insufficient sampling due to the study not being pathogenesis-oriented [19].

A previous study showed that 33.3% of OECs were cystic, and the remaining 66.7% were mainly solid, which was inconsistent with our findings [7]. Selection bias and misdiagnosis are the possible causes because research has found that 50 of 176 (28%) formerly diagnosed OECs are actually HGSCs [20]. The cystic component displayed homogeneous iso- or hyperintensity on T1WI in 82.6% (19/23) of OECs versus 4.3% of HGSCs and was another important differentiating feature. The iso- or hyperintensity on T1WI signifies bloody cystic content, which may imply OEC is derived from endometriosis [21].

In our study, almost all OECs and HGSCs demonstrated high signal in the solid component on DWI. The mean ADC value of the solid component was (0.979 ± 0.197) × 10^−3^ mm^2^/s in OECs versus (0.820 ± 0.112) × 10^−3^ mm^2^/s in HGSCs, and this difference was statistically significant. An optimal ADC value threshold of 0.971 × 10^−3^ mm^2^/s yielded a sensitivity, specificity and accuracy of 55.0%, 94.0% and 88.2%, respectively, for differentiating OECs from HGSCs, In our study, the solid component in OECs was mainly mural nodules or papillary projections, which were reported to have relatively low malignant potential [22]. Histopathologically, the mural nodules or papillary projections had a loose structure with fewer tumor cells, a fibrous axis, and interstitial edema; this explains the higher ADC value of OECs [23]. In contrast, the solid component in HGSCs was mainly a solid mass that histopathologically contained substantial tumor cells resulting in restricted movement of water molecules and, consequently, a lower ADC value.

Approximately 1-2% of gynecological malignant tumor cases have two or more synchronous primary genital tract carcinomas [8]. Approximately 10% of women with ovarian cancer will be found to have synchronous endometrial cancer, and approximately 5% of women with endometrial cancer harbor simultaneous ovarian cancer [24]. Synchronous OEC and endometrial cancer accounts for 50-70% of SPC [8,25]. On MR imaging, endometrial cancer of SPC often displayed superficial or no myoinvasion. In the present study, the synchronous primary endometrial carcinomas were found in 43.5% of OEC patients versus 4.3% of HGSC patients, which indicated high specificity for OEC. However, other studies found synchronous primary endometrial carcinomas in 16% of OEC patients [7].

Our study had several limitations. First, only imaging features of a limited number of patients were evaluated. Therefore, larger samples are necessary to confirm the value of these features for diagnosing OEC. Second, due to the retrospective design of our study, a selective bias was inevitable. Third, a correlation analysis of MRI features with pathology was not performed.

## Conclusions

In conclusion, our preliminary study demonstrates that a large, round or oval, mainly cystic mass with moderately enhanced mural nodules or papillary projections, a homogeneously iso- or hyperintense cystic component on TIWI, a relatively higher ADC value in the solid component and, commonly, SPC are features that help to differentiate OEC from HGSC. Although the diagnostic performance of any feature alone is not sufficient for diagnosis, the combination of the feature of a mainly cystic mass with mural nodules or papillary projections with any one of the following features: homogeneously iso- or hyperintense cystic component on TIWI, a relatively higher ADC value and SPC, yields high sensitivity, specificity and accuracy for identifying OEC.

## Abbreviations

OEC: Ovarian endometrioid adenocarcinoma
HGSC: High-grade serous adenocarcinoma
SPC: Synchronous primary cancer of the ovary and endometrium
